# Tuberculosis among migrant populations in the European Union and the European Economic Area

**DOI:** 10.1093/eurpub/cku208

**Published:** 2014-12-14

**Authors:** Anna Odone, Taavi Tillmann, Andreas Sandgren, Gemma Williams, Bernd Rechel, David Ingleby, Teymur Noori, Philipa Mladovsky, Martin McKee

**Affiliations:** 1 Department of Global Health and Social Medicine, Harvard School of Public Health, Boston, MA, USA; 2 Unit of Public Health, University of Parma, Parma, Italy; 3 European Observatory on Health Systems and Policies, European Centre on Health of Societies in Transition, London School of Hygiene and Tropical Medicine, London, UK; 4 European Centre for Disease Prevention and Control, Stockholm, Sweden; 5 LSE Health, London School of Economics and Political Science, London, UK; 6 Centre for Social Science and Global Health, University of Amsterdam, Amsterdam, The Netherlands

## Abstract

**Background**: Although tuberculosis (TB) incidence has been decreasing in the European Union/European Economic Area (EU/EEA) in the last decades, specific subgroups of the population, such as migrants, remain at high risk of TB. This study is based on the report ‘Key Infectious Diseases in Migrant Populations in the EU/EEA’ commissioned by The European Centre for Disease Prevention and Control. **Methods**: We collected, critically appraised and summarized the available evidence on the TB burden in migrants in the EU/EEA. Data were collected through: (i) a comprehensive literature review; (ii) analysis of data from The European Surveillance System (TESSy) and (iii) evidence provided by TB experts during an infectious disease workshop in 2012. **Results**: In 2010, of the 73 996 TB cases notified in the EU/EEA, 25% were of foreign origin. The overall decrease of TB cases observed in recent years has not been reflected in migrant populations. Foreign-born people with TB exhibit different socioeconomic and clinical characteristics than native sufferers. **Conclusion**: This is one of the first studies to use multiple data sources, including the largest available European database on infectious disease notifications, to assess the burden and provide a comprehensive description and analysis of specific TB features in migrants in the EU/EEA. Strengthened information about health determinants and factors for migrants’ vulnerability is needed to plan, implement and evaluate targeted TB care and control interventions for migrants in the EU/EEA.

## Introduction

Tuberculosis (TB) is a major public health concern. In 2013, there were an estimated 8.9 million (range 8.6–9.4 million) incident cases of TB globally, corresponding to 126 cases per 100 000 population. Moreover, TB was responsible for 1.5 million deaths.^1^ The greatest numbers of TB cases occur in low-income settings, predominantly Asia (56%) and Africa (29%).^1^

The World Health Organization (WHO) estimates that 4% of TB cases in 2013 occurred in the WHO European Region, with Eastern Europe particularly affected by the TB epidemic.^1^ Within the European Union/European Economic Area (EU/EEA), TB notification rates have been declining over the last decades, reaching 14.2 per 100 000 population in 2011.^2^ Despite this decline, specific subgroups of the population, such as homeless people, migrants, people living in urban settings and prisoners, remain at an increased risk of acquiring TB infection and developing active disease; this representing a challenge for TB control programmes.^3^^,^^4^ In particular, in many EU/EEA settings the contribution of cases among foreign-born individuals to the total TB burden is increasing each year.^5^ The pathways through which migrants are at higher risk for both transmission of TB infection and development of disease might include coming from high TB burden countries as well as being more exposed to socioeconomic and behavioural risk factors in their host countries. Migrants settled in host countries may also face legal, cultural, linguistic and socioeconomic barriers to healthcare that can delay TB diagnosis and limit access to health education and effective treatment.^6^^,^^7^

Few data are available on the TB burden in migrant populations in the EU/EEA. However, understanding the diverse health needs of migrants is becoming increasingly important, not least due to the rising proportion of migrants in the EU/EEA. From 1990 to 2010, the proportion of foreign-born individuals in the EU/EEA increased from 6.9 to 9.7% of the total population.^8^ In 2011, it is estimated that 48.9 million foreign-born residents were residing in the EU, with 32.4 million born outside it.^8^ To address the data gaps on infectious diseases among migrant populations, in 2012 the European Centre for Disease Prevention and Control (ECDC) commissioned a report titled ‘Key Infectious Diseases in Migrant Populations in the EU/EEA’. This article is based on that report and collects, critically appraises and summarizes the best available evidence on the burden of TB in migrant populations in the EU/EEA. Specific objectives are: (i) to estimate the burden of TB in foreign-born populations when compared with native populations in EU/EEA countries based on notification data, highlighting geographical patterns and time trends; (ii) to establish the burden of TB in migrants by gender, age group and country of origin as well as other relevant subgroups and (iii) to identify limitations of the available data and information gaps.

## Methods

To meet the specified aim and objectives, we retrieved data using three methods: (i) a comprehensive review of the literature, (ii) analysing relevant data from The European Surveillance System (TESSy) and (iii) evidence provided by infectious disease and migration experts at an ECDC meeting on migrant health held in 2012.^9^

### Comprehensive literature review

The literature search was conducted using Medline, Web of Science and The Cochrane Library as well as 18 websites of key organizations. It sought literature published up to September 2012. Additional records were retrieved through the citations of papers retrieved and consultation with experts in the field. Articles were considered for inclusion if they: (i) were descriptive and analytic observational studies, experimental studies, narrative reviews, systematic reviews and meta-analyses, guidelines or policy documents; (ii) were published after 2006; (iii) were published in English; (iv) included data from the EU/EEA. The search strategy was built using a combination of key words for the three main axes of the research question: (a) the selected disease: TB; (b) the study population: migrant populations; (c) study setting: the EU/EEA. For the PubMed search, free text terms were combined with Medical Subject Heading (MeSH) terms. Eligibility assessment for inclusion was performed in a two-step process independently by two reviewers, by first screening titles and abstracts and then full-text papers. Disagreements between reviewers were resolved by discussion. Data relevant to answer the study questions (detailed in the ‘specific objective’ earlier) were extracted to a predefined form. High priority was given to recent nationally representative studies and large population-based studies. No minimum sample size was selected but small hospital-based studies were only included if no other data were available and their limitations in terms of generalizability were reported.

### Analysis of TESSy data

TESSy is a metadata-driven platform managed by ECDC to collect, analyse and disseminate data on 53 communicable diseases and conditions under surveillance. All EU/EEA-Member States report data to TESSy. For the purpose of this study, we extracted data on TB from the TESSy database—including notification data, demographic and clinical characteristics as well as data relevant to the migration status of affected cases. Migrant status was determined by extracting data on the ‘country of birth’ variable.^10^

Case-based TB surveillance data were extracted from TESSy on 2 February 2012. Data for the years 2000-10 were analysed. Differences in reporting between countries were analysed and, where the data allowed it, trends over time were described. The TESSy database was interrogated to conduct further analyses on migrant populations not available in the ECDC surveillance report^2^ and used to complement it. All analyses were carried out in MS Excel 2013.

### Evidence provided by infectious disease and migration experts at an ECDC meeting on migrant health

In October 2012, the ECDC and the Portuguese National Institute of Health Dr. Ricardo Jorge (INSA) co-hosted an expert meeting on infectious diseases and migration in Lisbon, Portugal. Infectious disease and public health experts from Canada, France, Germany, Greece, Italy, Luxembourg, Spain, UK and USA participated as country representatives. They provided detailed country-level data on infectious disease surveillance, notification and reporting systems for migrant populations. For the purpose of this study, data relevant to TB in migrants were retrieved and reported.

## Results

### TB surveillance and reporting in the EU/EEA: focus on migrant populations

Since 2008, the collection and analysis of TB surveillance data in Europe has been conducted jointly by the ECDC and the WHO Regional Office for Europe (WHO/Europe). Data are collected from national surveillance institutions,^10^ and data from EU/EEA countries are validated by ECDC.^4^ In 2011, 25 countries in the EU/EEA reported information on origin of TB cases by the place of birth of those affected (born in the country vs. being of foreign origin) and five used the classification of citizenship of the respective country (citizen vs. non-citizen).^2^ Denmark and the Netherlands reported the birth place of cases’ parents; in Denmark, the geographical origin for TB cases under 26 years of age is classified according to parents’ place of birth (e.g. native-born cases under 26 years of age whose parents were born outside Denmark are classified as foreign-born cases).^10–12^ Data completeness for geographical origin of cases varies widely between countries.^2^^,^^10^

Our analysis of the TESSy data showed that up to 2001 very few countries reported case-based data on origin of TB cases to ECDC. After 2002 an increasing number of countries started to report on origin of TB cases; in the 2002-10 period, origin was known for nearly 80% of the TB cases.

### Burden of TB in the EU/EEA: focus on migrant populations

In the EU/EEA in 2010, 54 111 (73.1%) of the 73 996 notified TB cases were from the EU/EEA (referred to as ‘native’), 1284 (1.7%) were of unknown origin and 18 601 (25.1%) were of foreign origin.^11^ The percentage of foreign-origin cases ranged from 0.2% in Romania to 85.8% in Sweden (table 1).^11^ In six countries (Cyprus, Iceland, Malta, the Netherlands, Norway and Sweden), the percentage of foreign-born subjects among total TB notifications was >70%, whereas in 11 countries it was >50% (table 1).^12^ Of all EU/EEA countries, only Lichtenstein did not provide information on the origin of TB cases. Overall, the proportion of foreign-origin cases has been increasing since 2001.^12^ In 2010, the proportion of foreign-origin cases among total TB notifications in the EU/EEA was slightly higher than in 2008 (22.4%)^13^ and 2007 (21%).^4^ The overall decrease of TB cases observed in recent years has not been reflected in migrant populations. Only six countries (Belgium, Denmark, Estonia, Germany, Luxemburg and Slovenia) reported a decrease in TB cases of foreign origin, 11 countries reported an increase in cases and 10 countries reported no major changes over time.^10^

**Table 1:** Percentage of migrant TB cases (by country of birth or citizenship) in the EU/EEA, 2010

Country	Criterion	Foreign (%)	Total *N*
Austria	Citizenship	43.5	688
Belgium	Citizenship	54.6	1115
Bulgaria	Birthplace	0.1	2649
Cyprus	Birthplace	82.0	61
Czech Republic	Birthplace	17.3	678
Denmark	Birthplace	60.2	359
Estonia	Birthplace	17.6	329
Finland	Birthplace	32.1	327
France	Birthplace	48.3	5116
Germany	Birthplace	45.7	4330
Greece	Citizenship	47.2	489
Hungary	Citizenship	1.2	1741
Iceland	Birthplace	72.7	22
Ireland	Birthplace	40.0	427
Italy	Birthplace	55.7	3249
Latvia	Birthplace	6.6	934
Lithuania	Birthplace	2.4	1938
Luxembourg	Birthplace	58.6	29
Malta	Birthplace	78.1	32
Netherlands	Birthplace	73.5	1073
Norway	Birthplace	85.3	339
Poland	Citizenship	0.6	7509
Portugal	Birthplace	16.2	2626
Romania	Birthplace	0.2	21 078
Slovakia	Birthplace	1.8	439
Slovenia	Birthplace	23.8	172
Spain	Birthplace	32.0	7089
Sweden	Birthplace	85.8	675
United Kingdom	Birthplace	68.6	8483
Subtotal EU/EEA		25.1	73 996

### Country of origin and sociodemographic characteristics of foreign-born TB cases

**Figure 1:**
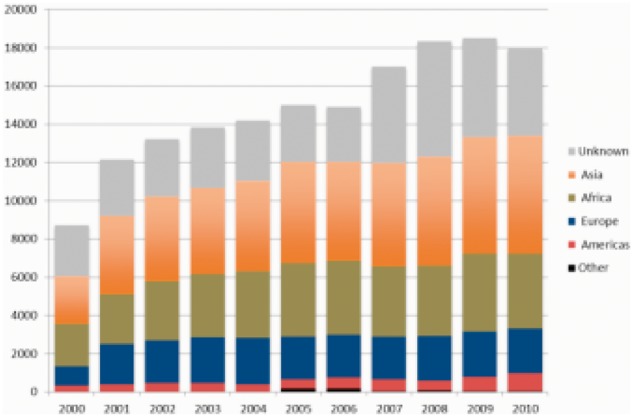
Notified migrant TB cases by country of birth or citizenship, EU/EEA 2000-10

Our analysis of the TESSy data showed that in 2010 the largest share of foreign-born TB cases were from Asia (34%), followed by Africa (22%) and other countries of the 53 countries of the WHO European region (13%). Of the cases coming from the WHO European region, 12% came from EU-Member States that joined prior to 2004, 56% came from EU-Member States that joined between 2004 and 2013 and 32% were from non-EU-Member States. The country of birth of foreign-born cases was unknown for 25% of migrant cases. Trends in the origin of foreign-born cases have been relatively stable over time, with the exception of an increase in the percentage of unknown country of birth between 2006 and 2008 (figure 1).

In a study from Italy, the distribution of the geographical origin of TB cases mirrored the distribution of the geographical origin of migrant populations in the country.^5^ In UK, 57% of foreign-born people with TB reported in 2010 were born in South Asia, 27% in Sub-Saharan Africa, with other regions accounting for lower percentages.^14^ Data from primary research in the EU/EEA show similar patterns. In the Netherlands, a recent study identified the main countries of origin of primary TB cases as Somalia, Morocco and Turkey^15^ which are among the main sources of the migrant population in the country. In Italy, the largest share of foreign-born TB cases come from the Middle East (36.6%) and Africa (21.7%) which are common countries of origin for migrants to Italy.^5^ Country experts at the Lisbon ‘Migrant health’ workshop reported that 8.4% of notified TB cases in Germany in 2010 with known country of birth were born in the Newly Independent States of the former Soviet Union (NIS: Armenia, Azerbaijan, Belarus, Georgia, Kazakhstan, Kyrgyzstan, Moldova, Russian Federation, Tajikistan, Turkmenistan, Ukraine, Uzbekistan and the three Baltic States Latvia, Lithuania and Estonia). Other foreign countries accounted for 36.2% of total TB cases.

TB cases in migrant populations tend to be in younger individuals than is the case with native citizens.^5^^,^^15^^,^^16^ More than half of TB cases of foreign origin notified in 2011 in the EU/EEA were in young adults (25-44 years) and ∼50% of cases of national origin were in the middle-aged (45-64 years) or elderly categories.^4^ As seen in the TESSy data, migrants with TB are ∼9 years younger (mean age: 37.5 vs. 46.3 years) than native sufferers, with this difference remaining stable in 2002-10 study period.

In both native and migrant populations, males are more frequently reported with TB. Overall, in the EU/EEA in 2010, the male:female sex ratio was 2.0:1 for native TB cases and 1.5:1 for foreign-born cases (table 1),^12^ the differences in ratios appears to be greater in migrants in some EU/EEA countries and less in others.

### TB incidence

**Figure 2:**
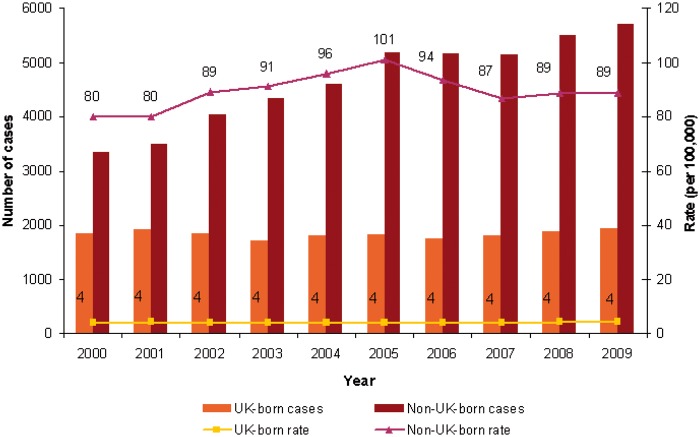
TB case reports and notification rates by place of birth, UK, 2000-09. Source: Health Protection Agency. Enhanced TB surveillance. Demography—TB trends by place of birth, England

TB incidence rates among migrants varied across the EU/EEA and in different study periods but, in the vast majority of EU/EEA countries, TB incidence in foreign-born populations is higher than in native populations.^15–17^ For example, as reported by country experts at the ‘Migrant Health’ Lisbon workshop,^18^ national surveillance data from France in 2009 showed that TB incidence in foreign-born subjects was nearly nine times higher than in subjects born in France (35.1/100 000 vs. 4.3/100 000). In UK, although the TB incidence in the foreign-born UK population rose from 75.5/100 000 population in 2000 to 81.6/100 000 in 2010, it remained stable at ∼4/100 000 in the UK-born population during the same period (figure 2).^17^ Data from regional and local studies conducted in the EU/EEA are consistent with national surveillance data.^5^^,^^16^ Analyses of TB incidence trends are not possible using the TESSy data, as no reliable population denominators for migrants are available for all EU/EEA-Member States.

### TB transmission by origin of cases

Data from the National TB surveillance system in the UK indicate that 77% of TB cases among foreign-born individuals are diagnosed 2 or more years after arrival in the host country.^14^ In contrast, surveillance data from France presented during the Lisbon workshop^18^ highlighted how TB notification rates in foreign-born subjects were higher in the first 2 years after entering France than later among all categories of migrants. Similarly, data from Spain reported that, in urban areas, around 50% of foreign-born TB cases were diagnosed with TB within the first 2 years after arrival.^19^ Such differences might be driven by different underlying TB burdens in countries of origin, as well as differences in migrants’ networks and exposures to social and behavioural risk factors in host countries. Several studies from Spain, Italy, Belgium and the Scandinavian region used both traditional epidemiologic approaches and molecular techniques to study the transmission dynamics and cluster characteristics of TB cases by country of origin.^16^^,^^19–24^ There is much heterogeneity among these studies, showing various degrees of transmission from foreign-born to native-born populations. The attribution of transmission in mixed clusters of native-born and foreign-born varies greatly between studies and countries. In some studies, the reported probability of TB transmission from foreign-born to native subjects in mixed clusters was low.^16^^,^^24^^,^^25^ For example, data from Germany show that, in mixed clusters, the probability that cases will be in foreign-born subjects was estimated at 18.3%.^16^ Some authors reported no evidence of significant TB transmission between native citizens and migrants.^16^^,^^25^ In particular, a large national-representative study conducted in Denmark showed that TB transmission was 2.5 times more likely to occur from Danes to migrants than vice versa.^25^ Data from Spain estimated that, in mixed clusters, index cases were foreign-born in 50% of cases.^19^

### Site of TB disease by origin of cases

Evidence from the UK shows extra-pulmonary TB to be associated with being of foreign-origin.^26^ In UK in 2010, 54% of foreign-born TB cases had extra-pulmonary TB while this percentage was only 31% in UK-born TB cases.^14^ Surveillance data also indicate that extra-pulmonary-TB is more frequent in foreign-born subjects several years after entry to the host country.^14^ In the Netherlands, notification data for the period 1993-2001 showed that non-Dutch nationals were more likely to have extra-pulmonary TB.^27^ In particular, during the study period, the absolute number of Dutch extra-pulmonary TB cases decreased (rate ratio per year: 0.96, 95% CI: 0.94–0.98), whereas the absolute number of non-Dutch extra-pulmonary TB cases increased (rate ratio per year: 1.06, 95% CI: 1.04–1.07).^27^

### Drug resistance by origin of cases

Multi-drug resistant (MDR) TB represents a major public health concern in Eastern Europe and Central Asia.^28^^,^^29^ Findings from the literature review showed no clear pattern in risk of drug resistance between foreign-born and native TB cases. The EU/EAA, ECDC/WHO surveillance data for the year 2010 reported lower percentages of MDR-TB in foreign-origin than in native cases (3.1% vs. 6.8%, respectively).^11^ Data from our TESSy analysis suggest higher proportions of MDR-TB in TB cases born in Northern Europe (13.9%), followed by Central Asia (12.0%), Eastern Europe (10.7%) and Eastern Asia (3.9%) with a mean MDR-TB percentage for all regions of 5.3%. However, there are differences in data completeness: resistance is unknown in 33-35% of cases from Northern Europe and Central Asia, and in 80% of cases coming from Eastern Europe. Few large nationally representative studies have specifically addressed the issue of MDR-TB in migrant populations in EU/EEA countries. Most available data are from hospital-based studies reporting data by country of origin. The majority of foreign-born patients with MDR-TB in the EU/EEA are from the former Soviet Union (FSU). In Germany, of the 184 MDR-TB patients identified through the network of hospitals participating in the Tuberculosis Network European Trials group, 80.2% were from FSU.^30^ In Finland, among all culture-verified incident MDR-TB cases diagnosed between 1994 and 2005; 73.7% were of foreign origin, mainly from Russia and Estonia.^10^^,^^31^

### HIV co-infection by origin of cases

Our analysis of TESSy data found that HIV status was unknown in 97% of foreign-born and 89% of native TB cases for the 2000-10 period. In 2010, HIV status was known in 27% of native and 4.7% of foreign-born cases. A 2011 survey attempted to assess current practices for monitoring HIV-TB co-infection in the EU/EEA.^32^ Eighteen out of 25 EU/EEA countries that responded reported collecting HIV status on individual TB cases. Of them, 10 countries reported having targeted programmes for TB testing for migrants in general, whereas four reported having targeted programmes for TB testing for asylum seekers, non-European migrants and Roma.^32^

**Figure 3:**
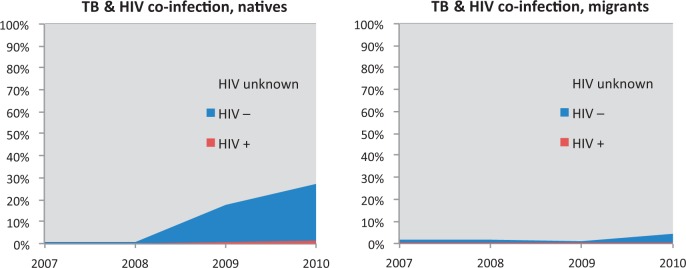
Co-infection of TB and HIV, by migration status—EU/EEA 2007-10 (TESSy analysis)

In 2010, EU/EEA aggregated data showed that among the 17 650 TB cases with known HIV status, 6.0% (range: 0–17.6%) were HIV positive.^11^ From our TESSy analysis, very limited case-based data on both HIV status and migrant status were available to allow for a disaggregated analysis. Thus, between 2000 and 2010, only 0.48% of native and 0.25% of foreign-born TB cases were notified as HIV positive (figure 3). During this period, the proportion of migrants with unknown HIV status was higher than the proportion in non-migrants (97% vs. 89%). In 2010, HIV status was known in only 4.7% of migrant TB cases compared with 27% in native-born TB cases.

Co-infection is especially common among certain marginalized populations, such as homeless people, injecting drug users and the subgroup of migrants. Foreign-born individuals represented >60% of notified TB-HIV co-infected cases in Belgium, England and Wales, France and the Netherlands (in England and Wales the percentage exceeded 80%) while lower percentages, of ∼30%, were reported in Italy and Spain.^33^ In Denmark, nearly 60% of TB-HIV co-infected cases were migrants.

### Paediatric TB by origin of cases

Between 2000 and 2009, 15.3% of paediatric TB cases notified in the EU/EEA were of foreign origin. This value was 29.2% in low TB incidence countries and 0.6% in high TB incidence countries.^34^ Overall, these percentages are lower than in the general population data previously presented. In 2010, the percentage of paediatric TB cases of foreign origin had increased to 19%.^11^ Only Norway, Sweden and Austria reported a higher number of foreign-origin TB cases than native-origin TB cases among children <15 years.^10^

Data on various aspects of TB in children are available from some EU/EEA-Member States, including Sweden, Denmark, UK, Spain and Greece.^35–37^ In Stockholm, 61% of laboratory-confirmed TB cases among children notified between 2000 and 2009 were from high TB burden countries and 25% were born in Sweden but from parents from such countries.^36^ Similar figures were reported from Denmark.^37^ In UK, 30% of TB cases notified between 1999 and 2006 in people aged <16 years were born outside UK.^35^ In London, the figure was higher (47.6%)^38^; the most common countries of origin were Somalia, Pakistan, India, Zimbabwe and Philippines. TB notification rates in children born outside UK was 37.3/100 000 (compared with 2.5/100 000 UK-born children). In Spain, the percentage of foreign-born subjects among paediatric TB cases increased from 2% in 1978-87, to 6% in 1988-97 and 46% in 1998-2007.^39^ Extra-pulmonary TB has been reported as more frequent among paediatric TB cases of foreign origin in some settings^37^ but not in others.^35^ As clinical symptoms of disseminated and extra-pulmonary TB forms may be less specific, this can negatively impact diagnostic delay among children.^40^ Furthermore, laboratory and radiological diagnosis can be challenging in the paediatric population. As a consequence, TB in children is usually identified though screening and active case finding (contact tracing) rather than following clinical manifestations. Children from high TB countries are at risk of acquiring infection before arrival in the host country.^36^ In UK, the median period between arrival in UK and TB diagnosis in migrant children is 2 years. Data from Spain and Denmark showed that when TB is acquired in the host country, most transmission occurs within the household^37^^,^^39^; however, in recent years, in these countries and in Sweden, index cases are increasingly being identified among friends and people in charge of taking care of children.^36^^,^^39^

## Discussion

To our knowledge, this is the first study to use multiple sources of data—including the largest available European database on infectious disease notifications—to assess the burden and specific features of TB in migrant populations in the EU/EEA.

Overall, in the EU/EEA, the share of foreign-born subjects among notified TB cases is increasing; this percentage is higher in Scandinavian and Northern EU/EEA countries and lower in Eastern EU/EEA settings. This relative rise of TB cases in foreign-born individuals is due to both a real increase, as well as attributable to the sizeable drop in native cases in certain countries.^2^ In addition, an increase in the absolute number of TB cases should be interpreted in the context of the global migration flows between different countries (Supplementary table S1). TB incidence rates are higher in foreign-born populations as compared with native populations. However, it is important to note that interpretation of TB incidence rates in migrants remains challenging.^5^ Unfortunately, migration statistics do not include irregular migrants and thus denominators may be underestimated. Moreover, as ECDC does not have access to migrants’ denominators figures (neither total, nor country of origin-specific denominators) surveillance data reported by the ECDC do not include incidence rates by geographical origin of cases.^10^

The country of origin of migrants might also impact on the TB burden in selected settings. For example, the UK, in contrast to other countries with high immigration rates, hosts many migrants from high TB burden countries (10% of migrants in the UK come from countries with a TB incidence ≥250/100 000) which may explain the increasing trends in TB incidence reported in the country. In other countries that have experienced progressively decreasing TB notification rates, such as Germany, Italy and Spain, the greatest share of migrants come from countries with relatively low TB incidence.

Foreign-born TB cases are younger when compared with native cases. This is mainly due to the different age structure of migrant and native populations in EU/EEA, but might also be linked to the different natural history of TB in the two populations.

No clear pattern emerged when analysing TB transmission by origin of case. It is likely that the disease develops through different pathways in foreign-born and native subjects. For migrants, clinical disease can come about through reactivation of infection acquired in the country of origin or through recent infection acquired in the host country. Furthermore, in some cases, infection in migrants is acquired during visits to the home country. On the contrary, in elderly native subjects, the disease may be more attributed to reactivation of latent TB infection. No evidence was retrieved on TB transmission between native citizens and migrants, and fears that the presence of migrants might increase TB in native populations seem to be unjustified.^16^^,^^25^ As mentioned earlier, the pathways through which migrants are at higher risk for both TB infection transmission and TB disease are difficult to disentangle. To determine which risk factors are associated with TB among migrant populations would require detailed studies in every country using individual level data, for example a series of case control studies, which is beyond the scope of the current study.

Migrants are more frequently reported to have extra-pulmonary TB than the native population. No clear pattern emerged on drug resistance by migrant status, although MDR-TB is most frequently reported in migrants originating from high MDR-TB burden settings. There is however a need to get a better understanding of drug-resistant TB among migrants. Reporting completeness of HIV status among TB cases is too low to derive meaningful conclusions, and improvements need to be made to collect better data on HIV status for both the native and migrant populations.

With regard to TB in children, foreign-born children account for a lower proportion among all paediatric TB cases (15.3%), when compared with the percentages of foreign-born reported in the overall population (26%).^14^ However, data on paediatric TB by origin of cases should be interpreted with caution, as surveillance data in most countries do not distinguish between children born in the host country of foreign-born parents from those born of native parents. This is a matter for concern because children of migrants may experience similar social, behavioural and environmental risk factors as foreign-born populations.^34^

Our study has limitations. These are mainly due to the heterogeneity of original studies in terms of study setting, study populations, data collected, methods applied and exposure and outcomes assessment which limited the potential of pooling estimates and findings. Moreover, the lack of data on denominators is a main limitation when assessing the burden of TB in migrant populations, as incidence estimates are often unreliable and tend to bias towards overestimations. In addition, only papers published in English were included in the literature review, which may limit the number of studies included from EU/EEA-Member States that do not routinely publish in English.

Available data on TB burden in migrant populations in Europe is incomplete, partly due to inconsistent surveillance and reporting systems in place in different countries and to the difficulties of properly tracing migrants in denominators when estimating incidence rates and trends. However, the collection and analysis of TB surveillance data in Europe since 2008 by the ECDC and WHO/EURO is an important step towards harmonization of national surveillance systems and data sharing. The TB case definition is applied consistently by EU/EEA-Member States in their surveillance systems and much relevant information on migration status is collected. In 2010, 29 EU/EEA countries reported data on the geographical origin of TB cases, with data completeness of 97.5%.

The efforts taken in recent years to strengthen and harmonize TB surveillance systems in the EU/EEA and capture migrant-specific information have led to a greater understanding of the association between migration and TB. Nevertheless, additional steps are still needed to strengthen national surveillance systems by harmonizing and improving data completeness. In addition, better data are needed on the extent to which health determinants and living conditions in the host country influence migrants’ vulnerability to TB. This information is of fundamental importance to better plan, implement and evaluate targeted TB prevention and control interventions in the EU/EEA. Priority must be given to addressing migrant health and ensuring that all individuals have access to prompt, high-quality TB care.

## Supplementary data

Supplementary data are available at *EURPUB* online.

## Funding

This work was supported by the European Centre for Disease Prevention and Control (ECDC).

*Conflicts of interest*: None declared.

**Key points**

- Migrant populations in the EU/EEA are at higher risk of tuberculosis infection and disease when compared with native-born populations. Among them, subgroups from high-TB burden countries are at greatest risk;
- The share of foreign-born cases (by country of birth or citizenship) among notified tuberculosis cases in the EU/EEA is 25%—ranging from 0.2% in Romania to 85.8% in Sweden—and has been increasing in the last decades;
- Foreign-born tuberculosis cases share different socioeconomic and clinical features when compared with native cases;
- Although the collection and analysis of tuberculosis surveillance data in Europe has been recently harmonized, available data on the tuberculosis burden in migrant populations in Europe is incomplete;
- Tuberculosis prevention and control programmes in the EU/EEA should target vulnerable populations, including migrants
